# Association between subthreshold depression and self-care behaviour in people with type 2 diabetes: a protocol for systematic review of observational studies

**DOI:** 10.1186/s13643-019-1084-7

**Published:** 2019-07-12

**Authors:** Monika Shrestha, Amal Al-Ghareeb, Fatimah Alenazi, Richard Gray

**Affiliations:** 1 School of Nursing and Midwifery, La Trobe University, Melbourne, Australia; 2 Nepal Diabetic Society, Kathmandu, Nepal; 3 Qassim University, Buraydah, Kingdom of Saudi Arabia; 4 University of South Australia, Adelaide, Australia; 5 University of Essex, Colchester, Essex, UK

**Keywords:** Subthreshold depression, Depression, Type 2 diabetes, Self-care behaviour, Systematic review

## Abstract

**Background:**

Depression is a common comorbidity in type 2 diabetes. Studies have consistently shown that major depression is associated with decreased diabetic self-care behaviour. People with subthreshold depression experience greater functional impairment, have a poorer quality of life and use health services more than those without depressive symptoms. Although subthreshold depression impacts self-care behaviour, the relationship between subthreshold depression and diabetes self-care behaviour has not been systematically reviewed. The objective of this systematic review is to determine the association between subthreshold depression and self-care behaviour in adults with type 2 diabetes.

**Methods:**

This protocol will follow the guideline of the Preferred Reporting Items for Systematic Review and Meta-Analysis Protocols 2015 (PRISMA-P-2015). A systematic search of literature will be conducted for observational studies reporting the association between subthreshold depression and self-care behaviour in adults aged 18 years or over and diagnosed with type 2 diabetes. Electronic databases including MEDLINE, EMBASE, PsycINFO, Emcare and CINAHL will be searched using predefined search terms. Title and abstract, full-text screening and data extraction of identified articles will be done by two reviewers independently. Discrepancies will be resolved by a third author. The methodological quality of the included studies will be assessed using The Joanna Briggs Institute (JBI) risk of bias tools. The review results will be presented in the form of narrative synthesis, and if sufficient studies are available and variability among the studies is low, a random effects meta-analysis will be done to quantify the result.

**Discussion:**

This review will synthesise evidence on the association between subthreshold depression and self-care behaviour in type 2 diabetic adults. The findings will be useful to researchers and policymakers to determine the most effective approach to overall diabetes management. The review will also identify research gaps in the current literature and provide direction for future research in this area of study.

**Systematic review registration:**

PROSPERO CRD42018116373

**Electronic supplementary material:**

The online version of this article (10.1186/s13643-019-1084-7) contains supplementary material, which is available to authorized users.

## Background

Diabetes is a complex and progressive metabolic disorder that affects approximately 425 million people worldwide [1]. Depression is a common comorbidity in type 2 diabetes and affects about 10–30% of adults with diabetes [2]. Compared to the general population, the prevalence of depression is twice as common in people with type 2 diabetes mellitus [2–4].

Subthreshold depression is a less severe form of depression, that occurs when an individual experiences depressive symptoms that do not meet the diagnostic threshold for a major depressive disorder in regard to frequency, severity and/or duration of symptoms [5, 6]. Common in adults, the prevalence rate of subthreshold depression is between 2.9% and 9.9% in primary care attendees and between 1.4% and 17.2% in community settings [6]. Subthreshold depression, interchangeably referred to as subclinical, significant, subsyndromal depression or minor depression [6], has been reported to be more prevalent than major depressive disorder in people with diabetes [7]. A total of 34.3% of people with diabetes had subthreshold depressive symptoms at the baseline assessment of a diabetic follow-up study in Canada [8]. A cross-sectional study of 808 participants in China reported the prevalence of subthreshold depression in people with type 2 diabetes to be 11.6% [9]. Moreover, it is estimated that around half of those with type 2 diabetes mellitus will experience at least one episode of subthreshold depression over a 5-year period [8].

Although subthreshold depression has less severe symptoms than a major depressive disorder [10], it causes a wide range of adverse outcomes [11–13]. For example, people with diabetes and subthreshold depression have impaired quality of life [14] and have more difficulties in achieving metabolic control [15, 16]. Like major depression, subthreshold depression also increases the risk for diabetic complications [17–19] and mortality [20, 21]. Adults with diabetes and subthreshold depression are more at risk of work [22] and functional disability [19] compared to those without depression. In addition, people with subthreshold depression have an increased risk of developing a major depressive disorder in the future [23, 24].

Diabetes is a complex disease that requires regular medical care for its effective management [25]. Self-care behaviour is the activities undertaken by people to manage their disease [26]. A critical element of diabetes care, self-care or self-management behaviour plays a significant role in maintaining individuals’ health and well-being [27]. According to the American Association of Diabetes Educators, there are seven self-care behaviours that predict good health outcomes. These behaviours are healthy eating, being physically active, monitoring of blood glucose, taking medication, good problem-solving skills, risk-reduction behaviours and healthy coping skills [28]. Evidence suggests that individuals who are not compliant to self-care activities are more likely to have poor metabolic control [29]. Nonadherence with self-care can lead to micro- and macrovascular complications [30] and poor glycemic control [31]. Poorly managed diabetes can cause serious complications that require hospitalisation, use of medical services and increased burden on the health care system [32].

For a patient with depression, self-management can be particularly challenging [33]. A meta-analysis conducted by Gonzalez et al. [34] that included 47 studies and 17,319 patients suggested a significant relationship between depression and nonadherence to diet, medication, exercise, glucose monitoring, appointment keeping and foot care [34]. However, the impact of depression differed across different self-care activities with the most substantial effect on missed medical appointments [34]. A systematic review of 27 studies and 7266 people with type 2 diabetes concluded that depression was associated with lower adherence to dietary and physical activity [35]. Similarly, individuals with both major or minor depression in people with type 2 diabetes showed an impaired problem-solving ability [36]. Depression is also found to be associated with decreased diabetes knowledge [37] and poor participation in patient education programmes [38].

Reviews published to date have largely focused only on major depression and self-care behaviour. Despite the increasing interest in subthreshold depression and its significant impact in both clinical and community population, to the best of our knowledge, the relationship between subthreshold depression and self-care behaviour has not been systematically reviewed. Therefore, the objective of this systematic review is to assess the association between subthreshold depression and self-care behaviour in type 2 diabetic population.

## Methods

This protocol complies with the guideline of the Preferred Reporting Items for Systematic Review and Meta-Analysis Protocols 2015 (PRISMA-P-2015) [39]. The detail of the PRISMA-P checklist can be found in Additional file 1. This protocol will guide the review and any deviations while conducting the review will be reported including the reasons for the changes made in the method section of the final published manuscript. The review has been registered in the International Prospective Register of Systematic Reviews (PROSPERO) with the registration number CRD42018116373. The proposed review will be guided and reported in accordance with the Preferred Reporting Items for the Systematic Reviews and Meta-Analyses (PRISMA) statement [40].

## Eligibility criteria

The following inclusion criteria will be applied to all stages of the review: Title and abstract screening and full-text screening. The inclusion criteria are:

- Any observational studies (cross-sectional, case-control and cohort)
- Studies conducted in adults aged 18 years or over and diagnosed with type 2 diabetes
- Studies that reported on the association between subthreshold depression and any one’s diabetes self-care activities
- Studies published in English

Qualitative studies, review articles and non-peer-reviewed (grey) literature will be excluded. Although exclusion of grey literature may limit some of the valuable data, the validity of the non-peer-reviewed literature is difficult to be determined. The self-care behaviours that will be included in the study are healthy eating, being physically active, monitoring of blood glucose, taking medication and risk-reduction behaviours (smoking, foot care) [28]. There is currently no definite definition of subthreshold depression given by existing diagnostic criteria (e.g. DSM-V or ICD-10) for depressive disorders [5]. Also, there is an extensive diversity in the terminology used to label subthreshold depression and substantial variation in the definition of the case used to categorise subthreshold depression [6]. In our review, we will include all those studies indicating subthreshold depression which do not meet the diagnostic criteria for major depression (subthreshold depression, minor depression, subclinical depression and subsyndromal depression) [41].

## Data source and search strategy

A systematic search of databases including MEDLINE, EMBASE, PsycINFO, Emcare and CINAHL will be carried out to identify all relevant articles. Search terms related to “type 2 diabetes”, “depression” and “self-care” will be used. The databases will be searched using the various combinations of medical subject headings (MeSH) and keywords. Appropriate Boolean operators (“AND”, and “OR”), proximity operators (“ADJ”, and “N”) and truncation will be incorporated into the search strategy to cater for the different use of terms. In consultation with a medical librarian experienced in systematic review database searching, a comprehensive MEDLINE (Ovid) search strategy has been developed (see Additional file 2). Other databases will be searched, and the search strategy will be adapted accordingly.

## Study selection

Citations identified from different databases will be imported into the reference manager software programme EndNote X9 (40). After removing duplicates, citations will be imported into Covidence systematic review software (www.covidence.org). Covidence is a software package developed to help in the management of systematic reviews. Two reviewers (MS and FA) will independently screen titles and abstracts according to the eligibility criteria. A third reviewer (AG) will resolve any disagreements between MS and FA. Full texts of potentially relevant articles will then be retrieved and again reviewed by two reviewers. The final decision regarding the inclusion of the studies will be made based on a thorough review of the full articles and joint discussion with the review team. The study selection process and the reason for exclusion will be documented. The outcome of the screening process will be reported in a PRISMA flowchart [40].

## Data extraction

A data extraction form will be developed in consultation with the review team. The following information will be extracted from the included studies: citation, country of study, aim of the study, study population characteristics (age, gender), study design (cross-sectional, case-control or cohort), study setting (community or hospital), sample size, sample size calculation, sampling technique, data source (survey or secondary data), definition of subthreshold depression, measure used (subthreshold depression, self-care behaviour), analysis, confounder variables adjusted and key observation of the study. For each study that meets the inclusion criteria, data extraction will be done independently by two reviewers (MS and FA). Any discrepancy between two reviewers during the data extraction process will be resolved through discussion and if necessary, a third (AG) reviewer will be consulted for the final decision. Authors will be contacted through emails to obtain missing or other relevant information if needed.

## Quality assessment

The Joanna Briggs Institute (JBI) Critical Appraisal Checklist tool will be used to assess the quality of studies. The JBI has separate checklists for cross-sectional (8 criteria), case-control (10 criteria) and cohort studies (11 criteria). Ratings will be made for the following criteria: eligibility criteria, study population, sample size, exposure measure, exposure time, exposure level, exposure before the outcome, confounders, outcome measures, follow-up and statistical analysis. Each component will be rated yes, no, unclear or not applicable. The summary of the quality of each study will be given. The studies will not be excluded based on their quality appraisal. Two reviewers (MS and FA) will independently assess the quality of each study. Any disagreement will be resolved by discussion and consensus within the review team.

## Data synthesis and statistical analysis

A systematic narrative synthesis of the findings from the included studies will be conducted. Tables and narrative summaries will be used to present the study characteristics and findings of all the included studies. The relationship between subthreshold depression and each self-care behaviour including the overall self-care behaviour will be discussed.

If sufficient studies are available and variability among studies is low, a random effects meta-analysis will be undertaken to compute the pooled estimate. Although the studies may be comparable for pooling the effect sizes, there may be certain variations in effect sizes across the studies due to different populations and study characteristics [42]. So, a random effects model will be used to determine the association between subthreshold depression and self-care behaviour, with the assumption that the true effects are normally distributed [42]. Given that studies are comparable in other characteristics, all these effect sizes will be converted into a common index, log odds ratio by following the method described by Borenstein et al [42]. Odds ratio (OR) will be used as a measure of association, and an alpha level of 0.05 will be considered statistically significant. Heterogeneity across the studies will be assessed with the Cochrane chi-square (*χ*^2^) and quantified with the *I*^2^ statistics [43]. *I*^2^ is the proportion of total variation provided by between-study variation, and *I*^2^ values of 25%, 50% and 75% will represent low, moderate and high heterogeneity, respectively [44]. We will check the potential publication bias by visual inspection of a funnel plot. In addition, Eggers’s regression test will be also done to statistically check the asymmetry of the funnel plot.

## Discussion

Subthreshold depression is a common comorbidity among type 2 diabetic patients. The effects of major depression on different self-care behaviours including physical activity, diet, blood glucose monitoring and foot care have been clearly documented. In this review, we intend to determine the relationship between subthreshold depression and self-care behaviour among type 2 diabetic people. Assessing the association between subthreshold depression and diabetes self-care is a critical step in identifying the most effective approach to overall diabetes management. Identifying an association between subthreshold depression and self-care activities may imply a need to integrate subthreshold depression screening in the treatment of type 2 diabetic patient. The effective management of comorbid subthreshold depression in people with type 2 diabetes could potentially enhance self-care behaviour resulting in better glycaemic control among other benefits.

The inclusion of studies only in the English language, the exclusion of grey literature and not undergoing peer review of the search strategy might be a potential limitation of the review. The heterogeneity between studies regarding the terminology used to label and define subthreshold depression and measures used to screen subthreshold depression and self-care behaviour may potentially limit to deriving a conclusion on the association between subthreshold depression and self-care behaviour. The review is expected to identify research gaps in the current literature and provide direction for future research in this area of study.

## Additional files

Additional file 1: Preferred Reporting Items for Systematic Review and Meta-Analysis Protocols 2015 (PRISMA-P-2015). (DOCX 33 kb)

Additional file 2: MEDLINE search strategy. (DOCX 15 kb)

## Data Availability

Not applicable.

## Abbreviations

JBI: The Joanna Briggs Institute
PRISMA: Preferred Reporting Items for the Systematic Reviews and Meta-Analyses
PRISMA-P: Preferred Reporting Items for Systematic Review and Meta-Analysis Protocols
PROSPERO: International Prospective Register of Systematic Reviews
