# Supplementary material for: Association between subthreshold depression and self-care behaviour in people with type 2 diabetes: a protocol for systematic review of observational studies
Source: Syst Rev. 2019 Jul 12;8:167. doi: 10.1186/s13643-019-1084-7 (PMC6624905; doi:10.1186/s13643-019-1084-7)
Supplement: Supplementary file 2 — MEDLINE search strategy. (DOCX 15 kb) [file 13643_2019_1084_MOESM2_ESM.docx]

Medline Search strategy:

| **#** | **Searches** | **Results** |
| --- | --- | --- |
| 1 | Diabetes Mellitus, Type 2/ | 118106 |
| 2 | ((type 2 or type2 or type II or typeII or type two) adj3 diabet*).mp. | 167336 |
| 3 | (MODY or NIDDM or T2DM or TDM2).mp. | 24012 |
| 4 | ((late or adult* or matur* or slow or stabl*) adj3 onset adj3 diabet*).mp. | 3060 |
| 5 | ((non insulin depend* or noninsulin depend*) adj3 diabet*).mp. | 11757 |
| 6 | 1 or 2 or 3 or 4 or 5 | 172389 |
| 7 | DEPRESSION/ | 105027 |
| 8 | exp Depressive Disorder/ | 101016 |
| 9 | depressi*.mp. | 420023 |
| 10 | 7 or 8 or 9 | 420697 |
| 11 | Self Care/ | 30965 |
| 12 | (self care or selfcare or self manage* or selfmanage*).mp. | 48954 |
| 13 | 11 or 12 | 48954 |
| 14 | exp DIET/ | 256164 |
| 15 | ((diet* or food) adj2 (habit* or behavio?r)).mp. | 17858 |
| 16 | diet*.mp. | 688694 |
| 17 | 14 or 15 or 16 | 729699 |
| 18 | exp Exercise/ | 172122 |
| 19 | (exercise* or physical activit*).mp. | 394654 |
| 20 | 18 or 19 | 439988 |
| 21 | Blood Glucose Self-Monitoring/ | 5789 |
| 22 | ((monitor* or test*) adj blood glucose).mp. | 960 |
| 23 | 21 or 22 | 6413 |
| 24 | patient compliance/ or medication adherence/ | 69874 |
| 25 | ((complian* or adheren*) adj2 medication*).mp. | 23686 |
| 26 | 24 or 25 | 75368 |
| 27 | (foot adj2 care).mp. | 1439 |
| 28 | 13 or 17 or 20 or 23 or 26 or 27 | 1223927 |
| 29 | 6 and 10 and 28 | 1201 |
| 30 | limit 29 to english language | 1147 |
